# Correction: Spatially-varying inversion near grain boundaries in MgAl_2_O_4_ spinel

**DOI:** 10.1039/d0ra90071k

**Published:** 2020-07-02

**Authors:** Blas P. Uberuaga, Romain Perriot

**Affiliations:** Materials Science and Technology Division, Los Alamos National Laboratory Los Alamos NM 87545 USA blas@lanl.gov; Theoretical Division, Los Alamos National Laboratory Los Alamos NM 87545 USA

## Abstract

Correction for ‘Spatially-varying inversion near grain boundaries in MgAl_2_O_4_ spinel’ by Blas P. Uberuaga *et al.*, *RSC Adv.*, 2020, **10**, 11737–11742, DOI: 10.1039/D0RA00700E.

The authors apologise for the incomplete description of the MC simulations methodology in the original article and would like to take this opportunity to warmly thank Enrique Martinez of LANL for allowing the use of his code to perform these simulations.

In the sentence beginning “Both the MC simulations and the minimizations were performed…” on page 11739, the corrected sentence should read as follows: “Both the MC simulations and the minimizations were performed using an in-house code driving LAMMPS,^39^ all the details about the MC methodology are still appropriate.

The Royal Society of Chemistry apologises for these errors and any consequent inconvenience to authors and readers.

## Supplementary Material

